# GP^4^: an integrated Gram-Positive Protein Prediction Pipeline for subcellular localization mimicking bacterial sorting

**DOI:** 10.1093/bib/bbaa302

**Published:** 2020-11-24

**Authors:** Stefano Grasso, Tjeerd van Rij, Jan Maarten van Dijl

**Affiliations:** University of Groningen; DSM Biotechnology Center in Delft, the Netherlands; University of Groningen and the University Medical Center Groningen, the Netherlands

**Keywords:** protein subcellular localization prediction, prediction methods, homology-based prediction, sorting signals, Gram-positive, GP4

## Abstract

Subcellular localization is a critical aspect of protein function and the potential application of proteins either as drugs or drug targets, or in industrial and domestic applications. However, the experimental determination of protein localization is time consuming and expensive. Therefore, various localization predictors have been developed for particular groups of species. Intriguingly, despite their major representation amongst biotechnological cell factories and pathogens, a meta-predictor based on sorting signals and specific for Gram-positive bacteria was still lacking. Here we present GP^4^, a protein subcellular localization meta-predictor mainly for Firmicutes, but also Actinobacteria, based on the combination of multiple tools, each specific for different sorting signals and compartments. Novelty elements include improved cell-wall protein prediction, including differentiation of the type of interaction, prediction of non-canonical secretion pathway target proteins, separate prediction of lipoproteins and better user experience in terms of parsability and interpretability of the results. GP^4^ aims at mimicking protein sorting as it would happen in a bacterial cell. As GP^4^ is not homology based, it has a broad applicability and does not depend on annotated databases with homologous proteins. Non-canonical usage may include little studied or novel species, synthetic and engineered organisms, and even re-use of the prediction data to develop custom prediction algorithms. Our benchmark analysis highlights the improved performance of GP^4^ compared to other widely used subcellular protein localization predictors. A webserver running GP^4^ is available at http://gp4.hpc.rug.nl/

## Background

Subcellular localization (SCL) is a key element in the functional annotation of proteins, their use in biotechnology, and their potential as drug candidates or targets. Ideally, SCL should be determined experimentally. Unfortunately, however, this is time consuming, expensive and impractical due to the recent explosion in the numbers of whole-genome-sequenced organisms. For such reasons, multiple approaches to predict SCLs have been developed (extensively reviewed in [1–5]).

Given that the prediction of SCL always starts from the amino acidic sequence of a protein, and the desired output is a designated cellular compartment or the extracellular milieu, the presently available approaches can be categorized based on the method of SCL assignment: (1) physico-chemical properties of the protein, (2) detectable sorting signals and (3) homology and transfer of knowledge. Each approach has its own advantages and disadvantages but, additionally, there can still be different methods implemented within each category that have their own specific pros and cons [1–5]. In this paper, we address the most relevant aspects that should be taken into account and present a new protein subcellular localization meta-predictor for Firmicutes, named GP^4^, which is also suitable for Actinobacteria.

Historically, the physico-chemical properties of a protein were the first parameters employed to predict signal peptides (SPs) for protein export from the cytoplasm and SCLs. However, physico-chemical properties by themselves are nowadays considered too broad for obtaining accurate results. Instead, two other approaches are regarded as more promising. SCL prediction based on known sorting signals is probably the most suitable approach, as the detection of specific localization tags embedded within the amino acidic sequence is also what cells do to sort their proteins [1, 6, 7]. However, a sufficiently detailed understanding of protein sorting mechanisms in the organism of interest is necessary to identify these localization tags with bioinformatic tools for SCL prediction. On the other hand, homology-based methods infer SCL by transferring the annotation of the best hit of a BLAST search to the query protein [2–5]. This last method is frequently used to functionally annotate genomes, genes and proteins. Unfortunately, however, it was estimated that homology-based annotations in the Gene Ontology (GO) database as of March 2006 showed an error rate of 49%. In contrast, homology-independent methods resulted in estimated error rates between 13 and 18% [8]. Altogether, the combination of a low number [9, 10] and biased distribution [11, 12] of studied and annotated entries in protein databases has resulted in the percolation of erroneous annotations [13, 14]. Moreover, while the transfer of annotations may appear effective [15, 16], different similarity thresholds can heavily influence the outcome, and will lead to annotation errors in case of low similarity [10, 16–18].

In addition to the three aforementioned methods for SCL assignment, also hybrid methods have been developed, which exploit the strengths and compensate for the weaknesses of the combined approaches and algorithms. This hybrid category encompasses the most frequently used and reliable SCL predictors, such as PsortB [19], CELLO [20], pLoc-mGpos [21] or Proteome Analyst [22].

Given the apparent lack of rational design in protein function or structure, it is important to consider the easiness by which evolution re-uses sequences for novel scopes, nullifying the ‘from sequence to structure to function’, and thus localization hypothesis [10, 18, 23]. Consequently, only annotations whose primary information source is experimental should be regarded with a certain confidence. Other types of annotation should be considered with care [24] and have in extreme cases led to the propagation of mistakes [25, 26]. Yet, experimental verification of protein SCL is also not flawless, as there is always cross-contamination during cell disruption, and it is hard to separate living cells from dead cells and their debris that has been released into the extracellular milieu [27].

Due to the major differences in the cellular structures encountered among the three main kingdoms of life (Bacteria, Eukarya and Archaea), bioinformatics tools generally specialize in SCL predictions for one of these domains of life. Unfortunately, within the Bacterial kingdom, the most common subdivision used is between Gram-positive and Gram-negative bacteria. This distinction is based on the outcome of Gram-staining with crystal violet rather than the cellular architecture and, consequently, leaves space for misinterpretations [28, 29]. Given the different morphology, the possible SCLs to be predicted differ substantially. In Gram-positive bacteria as traditionally defined, there are four classical sub-cellular compartments, namely the cytoplasm, the plasma membrane, the cell wall and the extracellular space. A further fifth compartment has been named the inner wall zone [30], which includes the ‘periplasmic’ area between the plasma membrane and the cell wall. However, to date, the inner wall zone has not been considered by SCL prediction tools. Despite an overall agreement on the different SCLs, there is little consensus amongst the different prediction tools about which proteins should be included in each compartment and the respective terminology [28]. Some proteins are unequivocal regarding their SCL, both from the computational and experimental points of view. Other proteins pose challenges since they may be experimentally found in multiple compartments, or may have been identified in SCLs that contrast with their *in silico* predicted SCLs. Additionally, some compartments can either be further subdivided or may be ‘atypical’, as exemplified by fimbriae, pili or spores, which do in fact possess their own peculiar subdivision (e.g., basal body, spore coat, cortex and core).

A crucial aspect in SCL prediction is its scope, or the origin of the query sequence. This can relate to a wild-type protein from a known or novel organism, or to a synthetically designed protein. Although this issue has been theoretically addressed [31], the latter category has never been thoroughly investigated. This may relate to the, thus far, limited needs to predict SCLs for synthetic proteins, but the design and realization of synthetic organisms is becoming more common and will probably increase in the future [32]. To properly address this kind of synthetic proteins, it is important to notice how they may be decontextualized from their original source or environment, *i.e.,* the original organism. In such cases, it may be misleading to directly assign the SCL retrieved from its closest wild-type homologue to the query sequence.

Lastly, a key aspect demanded by all users is the ease of interpretation of SCL predictions [7, 31]. Here, one also needs to consider context information that cannot be submitted with the query sequence, *e.g.,* the investigated species and its peculiarities or the applied design. One option to solve this dilemma is to increase the customizability and flexibility of the prediction tool, thereby allowing the user to include tailored options.

**Figure 1:**
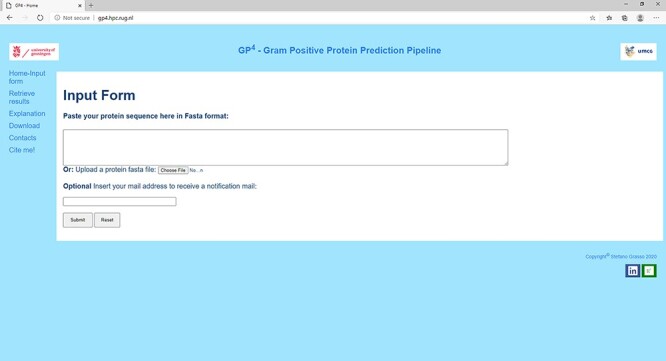
Homepage of the GP4 webserver. Input can either be pasted into the text box or uploaded as a file, both in fasta format. Optionally, it is possible to provide an email address to which the link with results will be sent. The interface is kept simple and no settings options are necessary. Results are stored for 7 days and can be retrieved at any moment through the specific page.

Taking into account so many aspects of SCL prediction is challenging, and multiple solutions with different pros and cons are possible. Here, we present a basic prediction pipeline for Gram-positive organisms called GP^4^ (Gram-Positive Protein Prediction Pipeline). In brief, GP^4^ is based on already available tools for different aspects of SCL prediction, which mainly rely on sorting signals or motif detection. GP^4^ minimizes the usage of homology to avoid the aforementioned biases. GP^4^ is particularly suitable for Firmicutes, and it is also effective for Actinobacteria, although it cannot predict their outer membrane proteins. GP^4^ is available as a webserver with an easy and user-friendly interface at http://gp4.hpc.rug.nl/ (Figure 1). The only required input is a list of fasta amino acid sequences, which can also be submitted as file. Additionally, GP^4^ can be used as a standalone program, but only as a pipeline script to produce the relevant data with the implemented tools, to combine them and to return the final SCL prediction.

## Material and methods

### Rationale and general approach

GP^4^ assigns up to five SCLs, including the four ‘classical’ ones, namely the cytoplasm, trans-membrane (TM), cell wall and extracellular. In addition, GP^4^ can also return lipoproteins as a result. Despite the latter not being a proper SCL, it was included in GP^4^ as a lipidic retention signal is often more informative with regard to protein sorting than the actual SCL. In contrast to other prediction tools, only integral membrane proteins with one or more TM α-helices are predicted by GP^4^ as membrane proteins. In contrast, peripheral membrane proteins associated with the cytoplasmic side of the membrane, which pose a semantic challenge in regard to their localization [7], are predicted to be cytosolic. Furthermore, we felt that membrane-bound proteins, like lipoproteins, should be classified on their own for the sake of clarity. To our knowledge, no other SCL prediction tool takes into account this issue, despite being discussed in literature [7, 31, 33]. Similarly, cell-wall proteins may be covalently attached to the peptidoglycan, or only transiently interact with it. In the absence of specific tools, we have tried to discriminate among these two possibilities. Furthermore, for extracellular proteins, GP^4^ provides the most likely secretion pathway based on detected signals, taking into account not only the main Sec and Tat pathways, but also alternative ones such as SecA2, the Wss route (i.e., the WXG100 secretion system, also called T7SSb), the flagellar export apparatus (FEA), the fimbrilin-protein exporter (FPE), and some lantibiotics and bacteriocins. Such aspects may be of lower relevance when analyzing bulk genomes for statistical purposes, but they may play major roles when analyzing specific protein candidates or engineered proteins. Lastly, it should be mentioned that GP4 fulfills the theorized properties of an expert system predictor [7, 31], based on its high interpretability, explanatory power, and its accountability for synthetically designed proteins.

### Software used

To develop GP^4^, multiple candidate tools were evaluated to cover all relevant aspects, including (i) detection of all possible secretion pathways; (ii) determination of TM topologies; and (iii) detection of domains, motifs, and repeats. For each aspect, the selection was further based on the reliability and efficiency of the various tools. Finally, usability and accessibility played a major role during selection. Considered criteria were the availability of downloadable or standalone versions, and limitations in the numbers or lengths of sequences that can be analyzed. Additionally, an overall parsimony approach was applied.

#### Signal peptides and secretion pathways

Detection and prediction of the correct secretion pathway is possibly one of the most challenging aspects of SCL prediction. The classical secretion pathway (Sec/signal peptidase I [SpI]) is the most studied and best characterized one, and thus, prediction of the respective SPs is most reliable. To detect these SPs, SignalP v. 4.1 [34], SignalP v. 5.0 [35], Phobius [36] and Predisi were exploited as they are specific for Sec SP detection. Additionally, also LipoP [37, 38], despite being mainly designed for lipoprotein SPs (Sec/signal peptidase II [SpII]) can help to determine the type of secretion pathway. Similar to LipoP, also SignalP 5 has the ability to detect lipoprotein SPs, as well as Tat SPs (Tat/SpI). To complement this ability of SignalP 5, also TatP [39] was integrated in the GP^4^ pipeline.

Unfortunately, tools to specifically predict other secretion pathways are currently not available. In particular, neither the signal peptides nor the proteins associated with protein secretion through ABC transporters, the SecA2 machinery, the FEA, FPEs, holins, the Wss route and any other non-classical secretion system (including moonlighting proteins) can thus far be predicted with dedicated tools. To at least partially overcome this limitation, InterPro signatures peculiar to these classes of proteins have been exploited in GP^4^ (see below).

#### Trans-membrane topology

TM helix detection is possibly one of the oldest predictable protein features. To detect them, TMHMM [40] has been used in GP^4^. Although TMHMM is a relatively old prediction tool, it is still considered efficient and reliable in its simplicity. To complement its ability to detect TM helices, topology predictions by Phobius were also taken into account.

#### Domains, motifs and repeats

For any other type of signal detection, signatures from InterPro [41] were used. InterPro collects and merges the entries from multiple databases and, additionally, manually curates them. This makes the various entries highly reliable. Nevertheless, given the fact that different methods and databases are used by InterProScan [42], different implemented signatures may have different levels of sensibility and sensitivity.

Manually curated lists of InterPro identifiers were created (Supplementary Table 1) for some of the main SCL targets, namely the secretion-associated signatures, Tat-associated signatures, lipoprotein-associated signatures and cell-wall associated signatures (in turn, subdivided into covalent bonds, non-covalent bonds and spore). In addition to those, and given the peculiar nature of some proteins, three additional lists were created to give a second, more detailed, level of SCL predictions: surface-associated signatures, pseudo-pilin- and fimbrilin-associated signatures and short secreted peptide-associated signatures (e.g., lantibiotics, bacteriocins or similar). Of note, even though most of the selected signatures are known and widely used to associate proteins with SCLs, they are not officially associated to any SCL (more precisely any GO compartment).

To exploit the full InterPro potential, lists of specific GO terms were created (Supplementary Table 1). During the motif and domain analysis through the GO compartment field, InterProScan may detect some that are officially associated to a specific SCL.

#### Other included tools

Lastly, ProtCompB [43], an online predictor for bacterial SCLs, was added to GP^4^ for additional support in the decision-making process. ProtCompB combines several prediction methods, namely: ‘neural networks-based prediction, direct comparison with bases of homologous proteins of known localization, comparisons of pentamer distributions calculated for query and database sequences and prediction of certain functional peptide sequences, such as signal peptides and transmembrane segments’ [43]. Thus, ProtCompB is fully complementary to the other aforementioned tools. In cases of doubtful decision-making, due to its highly reliable predictions [44, 45], ProtCompB can help in steering the results in the right direction.

#### Discarded tools

Despite the availability of additional tools for certain specific tasks (e.g., TM/SP discrimination or cell-wall-binding predictions), it was decided to discard them, because these tools could not analyze more than one sequence at a time, the tool size would not be compatible with most users’ machines, the outputs were graphical and would not be correctly parsed, or there were other usability issues.

### Dataset

The GP^4^ prediction algorithm was designed based on the current state-of-the-art knowledge about protein sorting in Gram-positive bacteria, in particular in *Bacillus subtilis*. Accordingly, in detail, testing was done based on the proteome of *B. subtilis* strain 168 (UP000001570).

Benchmark evaluation was performed with a test set, designated T1, of 374 proteins (summarized in Table 1; details of the dataset are presented in Supplementary Table 2). The dataset was built by retrieving from SwissProt [46], release 2020_02, all proteins belonging to the phyla of Firmicutes (id:1239) and Actinobacteria (id:1760) for which experimental evidence regarding the SCL was available for the respective species, and removing all proteins from in *B. subtilis* strain 168 (id:224308). This resulted in a set of 568 proteins. Afterwards, the redundancy was decreased to 25% identity by means of CD-HIT [47] (with standard settings), resulting in a total of 406 proteins. Finally, the dataset was further manually curated removing too short peptides or proteins whose SCL was classified as experimentally determined, but for which the published evidence was either poor or debatable. This yielded the final dataset of 374 proteins with known localization and at least one associated publication. Nevertheless, one should bear in mind that this curated dataset may still include some wrongly annotated proteins as there may be mistakes in the literature. This is exemplified by a public benchmarking dataset [21, 48] where, amongst proteins classified as cytosolic, there are also some secreted proteins, such as Q933K8 (now P9WJD9 [49, 50]) or P34020 [51]. Most likely, at the time of publication, the respective secretion system was yet to be discovered. This underscores the need to perform benchmarking always on the latest state-of-art datasets.

**Table 1:** Composition of the T1 protein dataset used for GP4 benchmarking

Localization	Number of proteins
Cell wall (CW)	58
Cytosolic (CYTO)	88
Extracellular (EXTRA)	133
Lipoproteins (LIPO)	9
Transmembrane (TM)	84
Multi-location (CW-TM)	2
Total	374

### Implementation

**Figure 2:**
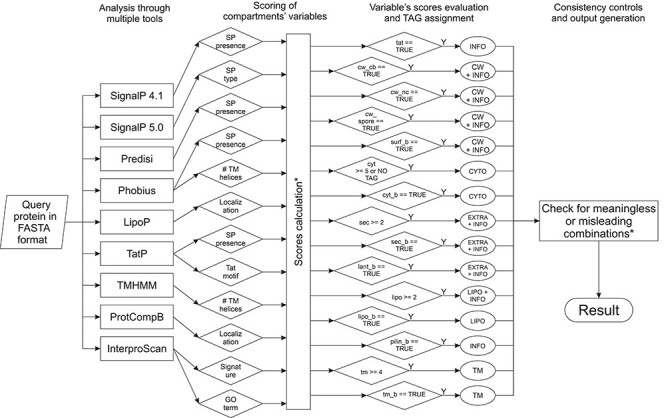
Summary of the prediction algorithm. The query protein is introduced in FASTA format and evaluated with multiple prediction tools. Based on the respective outputs, the values of 14 numerical and Boolean variables are calculated. If certain conditions or thresholds are met, then specific TAGs plus additional information will be assigned to the query protein. Lastly, a check of the different assigned TAGs is performed in order to remove redundant information. If certain TAGs are in conflict, ‘Unknown’ is returned as a result. ^*^, For the sake of clarity, particular details have been documented in Supplementary Table 3.

The prediction algorithm, summarized in Figure 2 (for more details see also Supplementary Table 3), was written in Python v. 3.6. It combines the outputs of the above-mentioned tools with a simple scoring system to return a putative SCL. Results from the different exploited tools are parsed and, depending on each tool’s output, scores for protein designation to the various compartments are increased or decreased. For each compartment’s score, there is a minimum threshold, which indicates the minimum amount of ‘evidences’ needed to assign an SCL TAG to a particular query protein. Additionally, if a sequence contains a compartment-related feature that indicates unequivocally a SCL, Boolean variables can override the scores. This has been implemented particularly in regard to InterPro signatures. Finally, through a series of simple ‘if-then-else’ conditionals, scores and Boolean variables are combined in order to assign one, or more SCL TAGs to a protein. Whenever a compartment’s score is higher than the set threshold, the respective SCL TAG is appended to the results for the query protein. The same has been implemented for Boolean variables. Normally, multiple TAGs can be assigned, if each of them individually meets its own requirements. Nevertheless, a last consistency check is performed: TAG combinations are evaluated and, in some cases, modified either because they are meaningless or potentially misleading. In such cases, either redundant information is removed (e.g., ‘EXTRA CW’ becomes ‘CW’, as cell-wall proteins are intrinsically extracellular) or, when in conflict, ‘Unknown’ is returned as a result (e.g., ‘EXTRA CYTO’ becomes ‘Unknown’, as there is no combination of signals that could lead to such a prediction). Additionally, ‘Unknown’ is also returned if no score reaches the required threshold. Next to the main SCL prediction, GP^4^ provides additional information, such as the secretion pathway used by a particular secretory protein, the signal peptidase cleavage site of putative SPs or the anticipated type of interaction with the cell wall. Such information is provided either paired with a specific SCL (e.g., detection of an LPXTG motif for covalent protein attachment to the cell wall) or independently (e.g., a Tat motif or pilin-like motif, as these motifs may suggest a final SCL, but do not completely determine it).

### Evaluation method

The test set T1 was used to evaluate the current prediction method and to compare it with other tools, namely PsortB [19] v. 3, LocTree3 [52], pLoc_bal-mGpos [21], Cello v. 2.5 [20] and BUSCA [53]. Proteins were analyzed, and predictions were used to calculate sensitivity, specificity, precision, accuracy and the Matthews correlation coefficient (MCC) for each class of proteins. These parameters are defined as follows:

\begin{equation*}\bullet\ \mathrm{Sensitivity:} \ \frac{TP}{TP+ FN} \end{equation*}

\begin{equation*} \bullet\ \mathrm{Specificity:} \ \frac{TN}{TN+ FP} \end{equation*}

\begin{equation*} \bullet\ \mathrm{Precision:} \ \frac{TP}{TP+ FP} \end{equation*}

\begin{equation*} \bullet\ \mathrm{Accuracy:} \ \frac{TP+ TN}{TP+ TN+ FP+ FN} \end{equation*}

\begin{equation*} \bullet\ \mathrm{MCC:} \ \frac{TP\times TN- FP\times FN}{\sqrt{\left( TP+ FN\right)\left( TP+ FP\right)\left( TN+ PF\right)\left( TN+ FN\right)}} \end{equation*}

TP, FP, TN and FN indicate true positives, false positives, true negatives and false negatives, respectively, for each localization. For a perfect set of predictions, the MCC value is 1, for a completely random prediction it will be 0 and for a perfect reverse prediction the MCC is −1. Lastly, the overall values for each tool were calculated as the weighted average between the various classes.

## Results and discussion

### Benchmark of SCL predictions

Benchmark comparative analyses with GP^4^ were performed using the majority of currently existing tools, normally returning alternate results [1, 53–59]. This was to be expected considering the fact that the composition of the train and test sets [60] and the relative internal amino acid sequence similarity levels [16, 20] will have a major impact on the outcome. The more the query sequence, or the species from which it is derived, is related to elements incorporated in the training set, the more precise the result returned by most tools will be. Even more so, this bias is present in homology-based prediction tools that rely on the presence and correct annotation of proteins within the respective database. Nevertheless, also tools based on the identification of motifs and signatures can have similar biases. This possibility should therefore be considered with respect to the following sections of this paper on the performance of GP^4^.

In addition to GP^4^, five other SCL predictors, namely PsortB v. 3 [19], LocTree3 [52], pLoc_bal-mGpos [21], CELLO v. 2.5 [20] and BUSCA [53] were benchmarked on the T1 dataset of 374 protein with known localization. The overall results are summarized in Table 2. LocTree3 turned out to be the best-performing tool with an overall MCC of 0.760. Nevertheless, it should be noted that LocTree3 is not able to discriminate between proteins from Gram-positive and Gram-negative bacteria. Additionally, it is not able to predict the cell wall as a compartment, classifying cell wall proteins simply as extracellular. Similarly, also BUSCA can only predict three compartments in Gram-positive bacteria, lacking the cell wall class. Similar to GP^4^, BUSCA is based on combining multiple tools for the detection of sorting signals, but, unfortunately, for Gram-positive bacteria, only SPs and TM helices are searched. Lacking many of the known bacterial sorting signals, BUSCA performs worse than LocTree3 with an MCC of 0.625, but it still provides useful information about the position of potential TM helices in detected SPs. Due to their simplicity, both tools can be an interesting choice to obtain a broad idea of the overall distribution of proteins. Nevertheless, in case of querying single proteins, or when a high level of precision is needed, more suitable tools are available. In particular, the here presented GP^4^ prediction tool, together with PsortB, pLoc_bal-mGpos and CELLO, is more suitable for a comparison as they include the four main SCLs of Gram-positive bacteria. Of note, GP^4^ provides an extra prediction result, namely ‘LIPO’ for lipoproteins, which does not in itself represent a sub-cellular compartment, but predicts with striking precision the membrane association of such proteins, resulting in an MCC of 1. Among these four tools, pLoc_bal-mGpos performed strikingly worse than expected with an overall MCC of 0.349. In contrast, CELLO proved to be better overall, but predicted only one cell-wall protein in the whole dataset, which lowered the overall scores. Given these results, it would make more sense to use simpler tools, like BUSCA or LocTree3, which can deliver better overall predictions. GP^4^, instead, performed slightly better than PsortB, with respective MCCs of 0.709 and 0.698. This outcome for PsortB was comparable with previous benchmarking analyses [57, 61]. It must be noted that, despite the similar MCC values, PsortB predicted 17.11% of the proteins as unknown, while for GP^4^, only 3.74% were predicted as unknown. More in detail, GP^4^ turned out the best-performing tool among the tested ones, with an MCC of 0.670, for cell-wall proteins (0.574 for PsortB; see also Supplementary Table 4). Instead, PsortB performed apparently better for extracellular proteins with an MCC of 0.736 (0.615 for GP^4^), but it must be noted that these values are influenced by the relevant difference in the rates of ‘unknown’ predictions for secreted proteins, namely 25.56% for PsortB versus 0.75% for GP^4^. This makes GP^4^ the best option to predict extracellular proteins in absolute numbers (i.e., taking into account the ‘unknown’ predictions by the two tools), as well as the most accurate. The improvement gained by GP^4^ for the prediction of extracellular and cell-wall proteins can probably be attributed to the detection of specific compartment-related domains.

**Table 2:** Summary of the GP4 benchmark analysis. The table summarizes the sensitivity, specificity, precision, accuracy and MCC for all benchmarked tools

Tool	Sensitivity	Specificity	Precision	Accuracy	MCC
GP^4^	0.781	0.909	0.823	0.88	0.709
PsortB	0.774	0.916	0.812	0.783	0.698
LocTree3	0.829	0.93	0.858	0.917	0.76
pLoc_bal-mGpos	0.524	0.827	0.529	0.773	0.349
BUSCA	0.714	0.908	0.823	0.793	0.625
CELLO v. 2.5	0.698	0.857	0.746	0.834	0.546

### Usage on modified proteins

**Figure 3:**
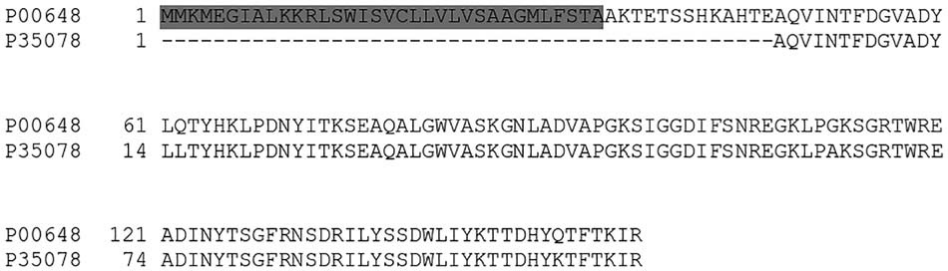
Sequence alignment of barnases from *B. amyloliquefaciens* and *B. circulans*. The barnase sequences were aligned with Clustal Omega. Gray shading marks the SP of the *B. amyloliquefaciens* barnase (P00648), which is absent from the barnase of *B. circulans* (P35078). Depending on the selected SCL prediction approach, P35078 would either be assigned as an extracellular protein since it belongs to a family of extracellular RNases, or as a cytosolic protein since it lacks a SP. Clearly, in absence of an appropriate SP, bacterial export of a protein with the P35078 sequence is unlikely.

If SCL prediction tools would be classified as text editors, PsortB would be considered as a WYSIWYM (what you see is what you mean), because it returns the SCL for the specific class of proteins. On the contrary, GP^4^ would be considered as a WYSIWYG (what you see is what you get) tool that predicts only what can be directly evinced from the actual query sequence. This is best exemplified by barnases, which are extracellular ribonucleases produced by various *Bacillus* species. As most secreted proteins, barnases do possess a Sec SP necessary for their export. The reference barnase was first discovered in *Bacillus amyloliquefaciens* (P00648) and possesses a SP and a propeptide according to SwissProt. Among homologous proteins with at least 90% identity, there is another barnase from *Bacillus circulans* (P35078). According to the annotation, this protein is 47 residues shorter and lacks a SP (Figure 3). The apparent lack of a SP is probably to be attributed to misannotation or low-quality sequence assembly, although we were not able to retrieve SP from the corresponding nucleotide sequence (data not shown). However, the protein in this form, i.e., without a SP, is unlikely to be secreted by a Gram-positive bacterium. Similarly, if we were to produce such a truncated protein in a heterologous strain, e.g., *B. subtilis*, it would hardly be secreted. Yet, all the tested prediction tools designated both barnases of *B. amyloliquefaciens* and *B. circulans* as extracellular proteins. This is formally correct for the regular barnases, but not for the barnase of *B. circulans* as it was annotated. On the contrary, GP^4^ predicted the *B. amyloliquefaciens* barnase to be secreted via Sec, while the truncated barnase of *B. circulans* was predicted to be cytoplasmic, as the amino acidic sequence lacks a SP. The latter approach is certainly favorable in the context of engineered organisms, but may be misleading when annotating wild-type genomes, and it can certainly not compensate for annotation errors. The latter can instead be managed by other approaches.

The main consequence of a WYSYWIG approach is the impossibility to predict protein sorting based on unknown or poorly characterized pathways. This should not be regarded as a negative aspect, but rather an incentive to improve the current knowledge and understanding of bacterial-sorting mechanisms and, at the same time, to develop novel and more precise tools to detect specific sorting signals embedded in the amino acidic sequence. In fact, in the present study, we show how SCL prediction based on detectable sorting signals can be more powerful than other approaches, regardless of the fact that many signals or motifs are still to be discovered or elucidated with respect to their function.

## Conclusions and future perspectives

In conclusion, the here presented GP^4^ tool seems to perform better than other SCL predictors, despite its intrinsic inability to predict SCLs for proteins that follow poorly characterized sorting pathways. In particular, GP^4^ should be appropriate for synthetic organisms, or organisms with little studied genomes. Furthermore, we consider GP^4^ the most widely-applicable tool for SCL predictions in Gram-positive bacteria. Due to its superiority in detecting extracellular and cell-wall proteins, it can probably help in the identification of novel targets for drugs against pathogenic Firmicutes and Actinobacteria. This is a consequence of its design, where prior knowledge on genomes or proteins is not necessary. On the other hand, the applicability of GP^4^ is limited by our overall understanding of protein sorting. For instance, GP^4^ was proven effective for SCL predictions in Actinobacteria, but it cannot predict the outer membrane proteins of this group. Only PsortB 3.0 can predict such outer membrane proteins, but only through a homology-based approach, as there is currently no other method or tool to detect this class of proteins. GP^4^ will thus predict Actinobacterial outer membrane proteins as secreted proteins, and it will remain a task for the user to perform further analyses to correctly assign their SCL. Altogether, we anticipate that experienced users will find GP^4^ applicable also for SCL predictions in other less-studied organisms, such as Tenericutes, but due to the current lack of proteins with known localization we have not tested this.

Particular attention should be attributed to the development of SCL prediction tools. While various tools have thus far been developed, none of them proved to be truly superior. Therefore, we advocate a paradigm shift in the development of SCL predictors. It was already known that meta-predictors perform better than single-purpose predictors [57, 62–64], because the meta-predictors exploit specific strengths while compensating for weaknesses of the individual tools. Yet, few advancements have been made in this direction, and no meta-predictor webserver for Gram-positive was thus far available. At least in prokaryotes, a stronger effort in developing sorting signal detectors, analogous to SignalP, should be made. In this regard also, the usability and parsability should be taken into account. This will lead to the creation of tools with standalone versions that do not rely exclusively on centralized webservers, and with standardized outputs that are easy to programmatically read and parse. These are prerequisites to develop better and more efficient meta-predictors, which could even be presented in a modular form with different tools being loaded, depending on the scope or source of the query. With these premises, the future development of SCL predictors may be brought to superior levels, as was achieved for the other two classes of functional annotation [65–69], and in other fields [70, 71].

**Key Points**

- Multiple methods for protein subcellular localization prediction are available, with different advantages and disadvantages depending on the origin of the query sequence.
- We propose to combine multiple single-feature predictors to mimic protein sorting within Gram-positive bacterial cells. This approach is knowledge-based and relies on our current understanding of prokaryotic biology, but not on prior knowledge of closely related organisms.
- GP^4^ is the first tool, which encompasses the capability to predict: (1) non-canonically secreted proteins; (2) lipoproteins, (3) cell-wall binding and interacting domains.
- When benchmarked against other subcellular localization prediction tools, the presented GP^4^ outperforms the other tools. In addition, GP^4^ provides extra information regarding the subcellular localization of query proteins, provides all data used to draw such conclusion and allows for a re-interpretation of results by experienced users.
- A webserver running GP^4^ is available at http://gp4.hpc.rug.nl/.

## Supplementary Material

Supplementary_Files_Descriptions_bbaa302

Supplementary_Table_1_R1_bbaa302

Supplementary_Table_2_bbaa302

Supplementary_Table_3_R1_bbaa302

Supplementary_Table_4_bbaa302

## Data Availability

The dataset used in this study, as described in the Dataset paragraph, is available as Supplementary Data. IDs to UniProt resources are referred to throughout the text.

## Code availability

In addition to the webserver, a standalone version of GP^4^ is available at https://github.com/grassoste/GP4_standalone.

## Notes

**Stefano Grasso** was a Marie-Curie fellow and a PhD student both at the University of Groningen and at DSM B.V. He received his M.Sc. from the University of Udine. His interests are at the edge between wet- and dry lab: synthetic biology, functional biology and -omics.

**Tjeerd van Rij** is a senior scientist at the DSM Biotechnology Center in Delft, the Netherlands. He obtained his PhD from Leiden University the Netherlands in 2006. His interests are in gene expression and protein secretion.

**Jan Maarten van Dijl** is a professor of molecular bacteriology at the University of Groningen and the University Medical Center Groningen, the Netherlands, since 2004. He earned his PhD degree in 1990. His research addresses fundamental and applied aspects of bacterial gene expression, protein secretion, virulence and antimicrobial drug resistance.
