# Supplementary material for: GP4: an integrated Gram-Positive Protein Prediction Pipeline for subcellular localization mimicking bacterial sorting
Source: Brief Bioinform. 2020 Nov 24;22(4):bbaa302. doi: 10.1093/bib/bbaa302 (PMC8294519; doi:10.1093/bib/bbaa302)
Supplement: Supplementary_Files_Descriptions_bbaa302 [file supplementary_files_descriptions_bbaa302.docx]

**Supplementary Table 1.** **Manually curated Interpro and Gene Onthology identifiers with respective descriptions.**

**Supplementary Table 2.** **Full description of the benchmark dataset T1.** Information was retrieved from Uniprot release 2020_02. The SCLs of all included protein were experimentally verified in at least one published study. In the last column (‘ACTUAL_LOC’) the most likely location used in the benchmark analysis is reported.

**Supplementary Table 3. Details of the GP^4^ prediction algorithm.** The Tab ‘Scoring of compartment variable’ provides the details of the contribution of the various outputs to the values of individual variables, as summarized in Figure 1 by the ‘Scores calculation’ process. For instance, the presence of a SP predicted by SignalP4.1 (column: ‘?_SigP4’; output value ‘Y’) will increase the variable ‘sec’ of 1 unit. The Tab ‘Conditions’ illustrate more clearly the condition needed to assign different TAGs and further information. The Tab ‘Not allowed combination’ provides the details of not allowed TAG combinations, either because they are biologically meaningless or highly questionable combinations, as summarized in Figure 1 by the ‘Check for meaningless or misleading combinations’ process.

**Supplementary Table 4.** **Full benchmark results obtained with the T1 dataset**. The Tab ‘Summary for classes’ shows the Sensitivity (SENS), specificity (SPEC), precision, accuracy and MCC for all classes and all benchmarked tools. The Tab ‘Absolute numbers’ presents the raw numbers for all tools and classes, including ‘Unknown’ predicted proteins, and TP, TN, FP and FN calculations. Correct classes as considered in this study are highlighted in green.
